# Pupal cocoons affect sanitary brood care and limit fungal infections in ant colonies

**DOI:** 10.1186/1471-2148-13-225

**Published:** 2013-10-14

**Authors:** Simon Tragust, Line V Ugelvig, Michel Chapuisat, Jürgen Heinze, Sylvia Cremer

**Affiliations:** 1 Evolutionary Biology, IST Austria (Institute of Science and Technology Austria), Am Campus 1, 3400 Klosterneuburg, Austria; 2 Evolution, Behaviour and Genetics, Biology I, University of Regensburg, Universitätsstr. 31, 93040 Regensburg, Germany; 3 Animal Ecology I, University of Bayreuth, 95440 Bayreuth, Germany; 4 Department of Ecology and Evolution, Biophore, UNIL-Sorge, University of Lausanne, 1015 Lausanne, Switzerland

**Keywords:** Social immunity, Sanitary brood care, Grooming, Hygienic behaviour, *Metarhizium* fungus, Formicidae

## Abstract

**Background:**

The brood of ants and other social insects is highly susceptible to pathogens, particularly those that penetrate the soft larval and pupal cuticle. We here test whether the presence of a pupal cocoon, which occurs in some ant species but not in others, affects the sanitary brood care and fungal infection patterns after exposure to the entomopathogenic fungus *Metarhizium brunneum*. We use a) a comparative approach analysing four species with either naked or cocooned pupae and b) a within-species analysis of a single ant species, in which both pupal types co-exist in the same colony.

**Results:**

We found that the presence of a cocoon did not compromise fungal pathogen detection by the ants and that species with cocooned pupae increased brood grooming after pathogen exposure. All tested ant species further removed brood from their nests, which was predominantly expressed towards larvae and naked pupae treated with the live fungal pathogen. In contrast, cocooned pupae exposed to live fungus were not removed at higher rates than cocooned pupae exposed to dead fungus or a sham control. Consistent with this, exposure to the live fungus caused high numbers of infections and fungal outgrowth in larvae and naked pupae, but not in cocooned pupae. Moreover, the ants consistently removed the brood prior to fungal outgrowth, ensuring a clean brood chamber.

**Conclusion:**

Our study suggests that the pupal cocoon has a protective effect against fungal infection, causing an adaptive change in sanitary behaviours by the ants. It further demonstrates that brood removal–originally described for honeybees as “hygienic behaviour”–is a widespread sanitary behaviour in ants, which likely has important implications on disease dynamics in social insect colonies.

## Background

Colonies of social insects have evolved collective disease defences to counteract the high risk of disease transmission within social groups (reviewed in [1,2]). This social immunity complements individual immune defences of all group members and comprises sanitary behaviours, use of antimicrobials and modification of interaction frequencies (e.g. [3-5]). Whereas adult colony members can display a variety of anti-pathogen defences, the brood depends on care by workers, particularly in the holometabolous social Hymenoptera (wasps, bees and ants), where eggs, larvae and pupae are largely immobile, in contrast to the hemimetabolous termites in which juvenile stages act as workers [6]. The cuticle of the larvae and pupae is not fully sclerotized and melanised [7], making the brood highly susceptible to infection with entomopathogenic fungi that enter their hosts by penetration of the body surface [8]. In social Hymenoptera, brood care therefore seems crucial to avoid fungal infection.

In ants, sanitary brood care by workers comprises mostly two complementary behaviours, 1) brood grooming, which reduces the pathogen load and germination ability of the pathogen [9], and 2) brood removal from the colony, termed “hygienic behaviour” [10]. Whereas grooming is a general response against pathogens in social insects (ants: [9,11], termites: [12,13]), hygienic behaviour is by definition restricted to the immobile brood of social Hymenoptera. It was originally reported from honeybees [2,14], and has recently been found also in ants [10]. It seems likely that brood grooming is a first line of defence against external pathogens, like the conidiospores of entomopathogenic fungi, while brood removal occurs as a second step, being triggered either by exposure or later by successful infection [10]. Whereas brood grooming may prevent infection of individual brood items, brood removal invariably leads to the death of the contaminated brood, but may reduce transmission to the healthy brood in the colony. In ants, where brood is placed together in joint brood piles [10], transmission risk among brood items is probably much higher than in wasps and bees, where each brood item is placed in an individual brood cell.

Most brood of ants is uncovered (“naked”), but in some species pupae are enclosed in a silk cocoon (larvae are always uncovered as they need constant feeding). The trait that larvae spin a silk cocoon upon pupation is remarkably variable in ants. It differs mostly among subfamilies but can also vary within subfamilies [15,16]. The function of cocoons remains debated, and ultimate explanations for the presence or absence of cocoons are still missing. It has been suggested that cocoons may protect the pupae either against 1) environmental fluctuation in temperature and humidity, 2) predators and parasitoids, or 3) microbial parasites and pathogens [17]. Given that brood in social insects is reared within the protected nest under controlled conditions [18], the first two mentioned functions may be of less importance, whereas a recent study, which describes how Attine ants cover their naked pupae in mycelia of their symbiotic fungus [15], discusses a possible protective function of the cocoon against pathogens in ants.

In this study, we test the hypothesis that the presence of a pupal cocoon may affect sanitary behaviours and fungal infection in ants. As our study system we chose five ant species and the entomopathogenic fungus *Metarhizium brunneum*, a natural pathogen of ants [19,20]. Upon contact with the insect cuticle, conidiospores of *M. brunneum* start to germinate and penetrate the cuticle to continue growth inside the host body. At high doses this eventually causes host death, after which fungal outgrowth of the corpse occurs [21]. We used *M. brunneum* to experimentally expose larvae and pupae of two ant species with naked pupae (*Linepithema humile* and *Crematogaster smithi*) and two ant species with cocooned pupae (*Lasius neglectus* and *Platythyrea punctata*), in order to analyse brood grooming and brood removal behaviour, as well as fungal infection patterns. The host species were chosen to represent different subfamilies of ants (Dolichoderinae, Myrmicinae, Formicinae, Ponerinae) with nesting ecology either directly in the soil or in rotten logs near the ground [22-25], therefore all being likely targets of soil-borne pathogens such as *M. brunneum*. Since between-species comparisons may be affected by phylogenetic constraints or other confounding species differences, we complemented this comparative approach with a within-species analysis, using a single, also ground-nesting, ant species (*Formica selysi,* Formicinae) in which naked and cocooned pupae co-occur within the same nest [26,27].

## Results

### Between-species comparison

#### Brood intake

Across species, a total of 72% of all presented brood was carried into the brood chamber within the first two days of the experiment. Except for *C. smithi*, which brought in more larvae (L) than pupae (P) (69% L, 44% P; χ^2^-test: χ^2^ = 8.186, d.f. = 1, P = 0.004), both brood types were retrieved at equal rates (*Li. humile*: 67% L, 51% P, χ^2^ = 1.699, d.f. = 1, P = 0.192; *La. neglectus*: 75% L, 75% P, χ^2^ = 0.037, d.f. = 1, P = 0.847; *P. punctata*: 100% L, 100% P, χ^2^-testing inappropriate due to 100% intake for both L and P). Brood intake was not affected by treatment, i.e. whether brood items had received a sham treatment or had been exposed to dead or live fungal conidiospores, in *Li. humile*, *La. neglectus* and *P. punctata* (Table 1; Cox mixed-effects model). Only *C. smithi* brought in fewer pupae treated with live fungus than dead fungus or sham control, with the latter being retrieved at non-significantly different rates (Wald-χ^2^ = 11.53, d.f. = 2, P = 0.003; pairwise comparisons: live fungus vs sham control: P = 0.002; live vs dead fungus: P = 0.005, dead fungus vs sham control: P = 0.086). Workers of all species placed all brood, irrespective of type and treatment, onto a single pile in the brood chamber and groomed the brood.

**Table 1:** Brood intake in the between-species comparison

	**Larvae**						**Pupae**					
	**Sham control (%)**	**Dead fungus (%)**	**Live fungus (%)**	**Wald-χ**^**2**^	**d.f.**	**P**	**Sham control (%)**	**Dead fungus (%)**	**Live fungus (%)**	**Wald-χ**^**2**^	**d.f.**	**P**
*C. smithi*	75	67	67	3.110	2	0.211	67	46	21	11.53	2	**0.003**
*Li. humile*	75	58	67	0.993	2	0.609	63	42	50	3.029	2	0.220
*La. neglectus*	75	75	75	0.270	2	0.874	75	75	75	0.208	2	0.901
*P. punctata*	100	100	100	< 0.001	2	1.000	100	100	100	< 0.001	2	1.000

Percentage of larvae and pupae (treated with either sham control, dead or live fungus) taken into the brood chamber by ant species with either naked (*C. smithi* and *Li. humile*) or cocooned pupae (*La. neglectus* and *P. punctata*). Statistics for brood intake rates originate from the Cox mixed-effects model. Significant *P* values (with α = 0.05) are denoted in bold.

#### Brood grooming

**Figure 1:**
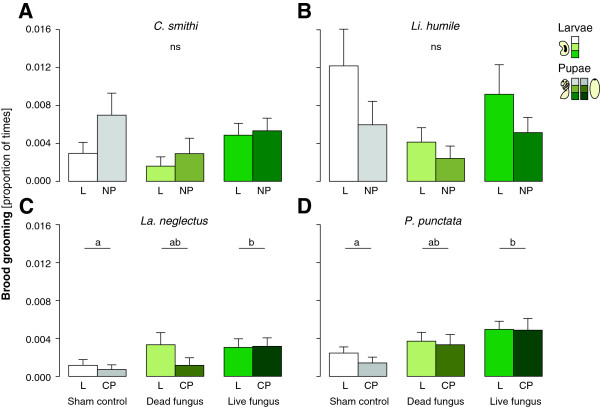
**Brood grooming in ants with naked vs cocooned pupae.** Grooming frequency (proportion of times grooming was performed on total observations) received by the brood (L: larvae, NP: naked pupae, CP: cocooned pupae) treated with either sham control (white to grey bars), dead fungus (light green tones) or live fungus (dark green tones) is shown for the two species with naked pupae, *C. smithi***(A)** and *Li. humile***(B)**, and the two species with cocooned pupae, *La. neglectus***(C)** and *P. punctata***(D)**. Larvae and pupae received equal grooming in all species. Only the two species with cocooned pupae **(C**,**D)**, but not the ones with naked pupae **(A**,**B)**, groomed live fungus-exposed brood more frequently than the sham control, with dead fungus-treated brood being groomed at intermediate rates. Mean + s.e.m. are depicted, different letters denote statistical differences at the significance level α = 0.05 (ns: not significant).

Workers of all four species overall groomed larvae and pupae at similar frequencies (Figure 1A-D; Linear Mixed Model, LMM, *C. smithi:* F_1,25_ = 1.156, P = 0.293, *Li. humile:* F_1,37_ = 2.936, P = 0.095, *La. neglectus:* F_1,40_ = 1.506, P = 0.227; *P. punctata:* F_1,42_ = 2.446, P = 0.125). The two species with naked pupae showed no significant differences in grooming frequency between sham-treated and dead or live fungus-exposed brood (Figure 1A,B; LMM, *C. smithi:* F_2,25_ = 3.166, P = 0.06; *Li. humile:* F_2,37_ = 2.168, P = 0.129). In contrast, the two species with cocooned pupae groomed live fungus-exposed brood significantly more than sham-treated brood, and showed a near-significant trend for higher grooming frequency of brood treated with live vs dead fungus (Figure 1C,D; *La. neglectus:* F_2,40_ = 3.683, P = 0.034; *P. punctata:* F_2,42_ = 6.309, P = 0.004; post hoc Tukey comparisons: live fungus vs sham treatment: P = 0.019 and P = 0.001, live vs dead fungus P = 0.134 and P = 0.076, dead fungus vs sham treatment P = 0.228 and P = 0.075 for *La. neglectus* and *P. punctata* respectively).

#### Brood removal

**Figure 2:**
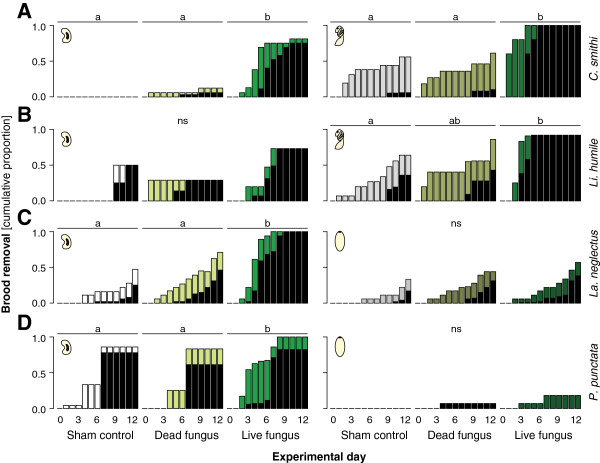
**Bood removal and fungal outgrowth in ants with naked vs cocooned pupae.** Cumulative proportion of brood removed from the brood chamber over the experimental period for the two species with naked pupae, *C. smithi***(A)** and *Li. humile***(B)**, and the two species with cocooned pupae, *La. neglectus***(C)** and *P. punctata***(D)**, presented for larvae (left) and pupae (right) depending on brood treatment (sham control: white to grey bars, dead fungus: light green tones, live fungus: dark green tones). The proportion of removed brood showing fungal outgrowth on each day is depicted in black. Live fungus-exposed larvae and naked pupae were removed significantly faster and at higher numbers than sham-treated and dead fungus-exposed brood in all species except *Li. humile* (ns for larvae and ns for naked pupae treated with dead vs live fungus). In cocooned pupae pathogen-exposure did not lead to increased removal compared to the two non-infectious treatments. Fungal outgrowth occurred in a high proportion of removed brood, several days after removal from the brood chamber. Cross-contamination by pathogen transmission from the live fungus-exposed brood to the other two treatments occurred across all brood types, leading to delayed fungal outgrowth also in originally sham-treated and dead fungus-treated brood. Different letters denote statistical differences at the significance level α = 0.05 for brood removal (ns: not significant).

Overall, 49% of all brood that was previously brought into the brood chamber was removed again over the twelve days of the experiment in the four ant species. The two species with naked pupae removed more pupae than larvae (*C. smithi*: 30% L, 63% P, χ^2^-test: χ^2^ = 7.148, d.f. = 1, P = 0.008; *Li. humile*: 29% L, 70% P, χ^2^ = 8.318, d.f. = 1, P = 0.004), whereas the two species with cocooned pupae removed fewer pupae than larvae (*La. neglectus*: 69% L, 41% P, χ^2^ = 7.322, d.f. = 1, P = 0.007; *P. punctata*: 72% L, 6% P, χ^2^ = 47.867, d.f. = 1, P < 0.001). Larval removal patterns depended strongly on treatment. Larvae exposed to live fungal conidiospores were removed earlier and at higher rates than larvae exposed to dead fungus or sham treatment, which was significant for all species except *Li. humile* (Figure 2A*-*D; Cox mixed-effects model: *C. smithi*: Wald-χ^2^ = 20.67, d.f. = 2, P < 0.001; *Li. humile*: Wald-χ^2^ = 1.385, d.f. = 2, P = 0.500; *La. neglectus*: Wald-χ^2^ = 29.03, d.f. = 2, P < 0.001; *P. punctata*: Wald-χ^2^ = 16.23, d.f. = 2, P < 0.001; post hoc Tukey comparisons: live vs dead fungus and sham control: all P < 0.05 and dead fungus vs sham control: all P = n.s., for *C. smithi*, *La. neglectus* and *P. punctata*). Similarly, in the two species with naked pupae, live fungus-exposed pupae were removed at higher rates than sham-treated pupae (Figure 2A,B; *C. smithi*: Wald-χ^2^ = 11.03, d.f. = 2, P = 0.004; *Li. humile*: Wald-χ^2^ = 8.721, d.f. = 2, P = 0.015; post hoc Tukey comparisons: live fungus vs sham control: P ≤ 0.013), and also than dead fungus in *C. smithi*, whereas *Li. humile* only showed a near-significant trend in this direction (live fungus vs dead fungus; *C. smithi*: P = 0.005, *Li. humile*: P = 0.073; dead fungus vs sham control for both species P ≥ 0.441). In contrast, treatment had no significant effect on pupal removal in the species with cocooned pupae (Figure 2C,D; *La. neglectus*: Wald-χ^2^ = 2.254, d.f. = 2, P = 0.324; *P. punctata*: Wald-χ^2^ = 0.689, d.f. = 2, P = 0.709). The general emerging pattern was thus that removal of larvae and naked pupae was increased after the live fungus treatment, whereas cocooned pupae exposed to live fungus were removed at equally low rates as cocooned pupae treated with either a sham control or dead fungus. Lastly, all species created a common dump pile outside of the nest where removed brood was placed.

### Within-species analysis

#### Brood intake

*F. selysi* workers brought 69% of all presented pupae into the brood chamber within the first two days of the experiment, with cocooned pupae being taken in at somewhat higher numbers (78%) than naked pupae (61%; χ^2^-test: χ^2^ = 3.96, d.f. = 1, P = 0.047). Brood treatment (sham control, dead or live fungus) did not significantly affect brood intake rates (naked pupae: 71% sham control, 67% dead and 46% live fungus, Cox mixed-effects model: Wald-χ^2^ = 5.539, d.f. = 2, P = 0.063; cocooned pupae: 83% sham control, 71% dead and 83% live fungus, Wald-χ^2^ = 1.838, d.f. = 2, P = 0.399).

#### Brood grooming

**Figure 3:**
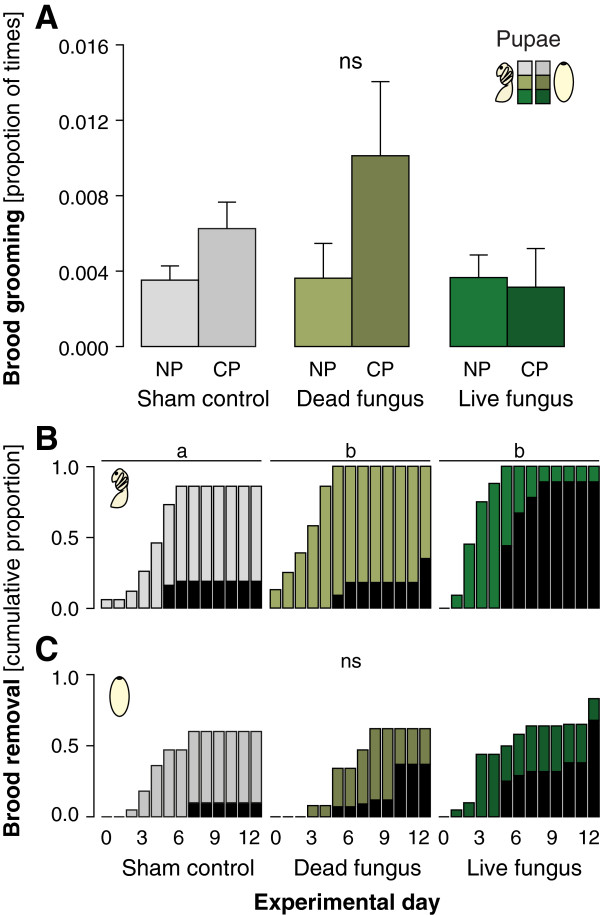
**Brood grooming, brood removal and fungal outgrowth in an ant with both, naked and cocooned pupae. (A)** Naked pupae (NP, lighter colours) and cocooned pupae (CP, darker colours) of *F. selysi* were groomed at similar frequencies (proportion of times grooming events occurred on total observations), and independently of brood treatment (sham control: grey tones, dead fungus: light green tones, live fungus: dark green tones). Means + s.e.m. are given. **(B**,**C)** Cumulative proportion of naked pupae **(B)** and cocooned pupae **(C)** removed from the brood chamber over the course of the experimental period, for each treatment (same colour scheme as in **(A)**). Black bars depict the proportion of removed pupae that showed fungal outgrowth on each day. Whereas fungus-exposed naked pupae were removed more quickly and more frequently than sham controls **(B)**, cocooned pupae were not removed differently according to treatment **(C)**. Different letters denote statistical differences at the significance level α = 0.05 level (ns: not significant) for grooming frequency **(A)** and for brood removal rates **(B**,**C)**.

Neither pupal type (naked vs cocooned; LMM, F_1,32_ = 1.842, P = 0.184) nor brood treatment (sham control, dead or live fungus; F_2,32_ = 1.144, P = 0.331) significantly affected grooming frequencies in *F. selysi* (Figure 3A).

#### Brood removal

*F. selysi* workers removed 54% of all pupae previously taken into the nest, thereby taking out fewer cocooned (43%) than naked (61%) pupae (χ^2^-test: χ^2^ = 5.383, d.f. = 1, P = 0.02). Brood treatment affected the removal of naked pupae (Figure 3B; Cox mixed-effects model: Wald-χ^2^ = 10.83, d.f. = 2, P = 0.004), but not of cocooned pupae (Figure 3C; Wald-χ^2^ = 1.421, d.f. = 2, P = 0.491). Naked pupae that were exposed to live fungus were removed significantly faster and in higher numbers than sham treated pupae (post hoc Tukey comparisons: P = 0.004), but not differently than dead fungus-exposed pupae (P = 0.235), which were also removed more than the controls (P = 0.018).

### Fungal outgrowth

We determined the infection status of all individual dying workers and brood items (which were colour-marked according to treatment) by observation of fungal outgrowth. Overall, only 0.6% (2/300; 1 *C. smithi* and 1 *La. neglectus*) of the workers from all five species died during the twelve days of the experiment from contracting an infection with *M. brunneum*. Over all species, brood with fungal outgrowth was mostly found outside the brood chamber (64%; 152/236) and only rarely inside (4%; 10/235; difference in location, χ^2^-test: χ^2^ = 95.289, d.f. = 1, P < 0.001). Fungal outgrowth was not restricted to the brood items that were experimentally exposed to live fungal conidiospores, but also occurred on sham-treated and dead fungus-exposed brood (inside: sham control: 4/10; dead fungus: 2/10; outside: sham control: 30/152; dead fungus: 36/152), indicating that the disease had been transmitted to approximately 22% of the previously healthy brood. Fungal outgrowth on these groups occurred with some delay compared to the originally exposed group (black bars in Figures 2 and 3B,C).

**Figure 4:**
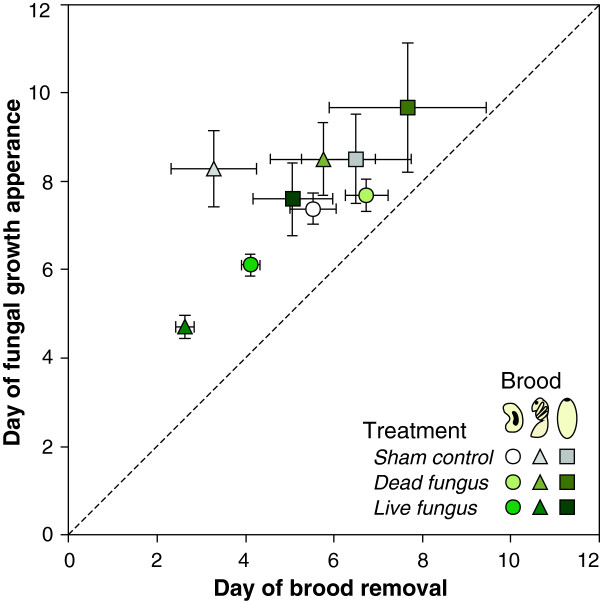
**Correlation between the timing of brood removal and fungal outgrowth.** For brood items with fungal outgrowth, the time of fungal growth was positively correlated with the time of removal from the brood chamber, and the removal by the ants preceded fungal outgrowth (demonstrated by all points lying above the diagonal). Whereas live fungus-exposed larvae and naked pupae were removed fast and showed early fungal outgrowth, the cocooned pupae cluster with the sham controls and dead fungus-treated brood. Means ± s.e.m. for all species per brood type and treatment are shown.

Across all study species, the brood with fungal outgrowth showed a positive correlation between the time of outgrowth and the time of removal from the brood chamber (Pearson’s correlation: R^2^ = 0.753; t = 3.026, d.f. = 7, P = 0.019), with removal always preceding fungal outgrowth (Figure 4). When comparing fungal outgrowth between brood types that were exposed to live fungal conidiospores, a significantly lower proportion of cocooned pupae showed fungal outgrowth compared to both naked pupae and larvae (larvae: 71%, naked pupae: 82%, cocooned pupae: 35%; χ^2^-test: overall: χ^2^ = 31.624, d.f. = 2, P < 0.001, pairwise posthoc comparisons: larvae vs naked pupae: χ^2^ = 0.727, d.f. = 1, P = 0.394, larvae vs. cocooned pupae: χ^2^ = 14.154, d.f. = 1, P < 0.001, naked vs cocooned pupae: χ^2^ = 14.448, d.f. = 1, P < 0.001, both being smaller than the Bonferroni-adjusted significance level α = 0.017; black bars in Figures 2 and 3B,C).

## Discussion

We found that the presence of a pupal cocoon affects sanitary behaviours and fungal infection in ants. Species from different subfamilies of ants showed consistent patterns depending on pupal type (naked versus cocooned), suggesting that the presence or absence of the cocoon may be a main predictor of the observed effects. This is further corroborated by the fact that similar patterns were found within a single species that simultaneously produces naked and cocooned pupae within the same nest. Still, as we have only covered four subfamilies of ants (one species each of Ponerinae, Myrmicinae, Dolichoderinae, and two species of Formicinae), the generality of this finding across the whole ant phylogeny remains to be tested.

Similar to previous studies [28,29], the ants in our experiment showed some differences in retrieval of the various brood types, likely as a consequence of chemical, morphological, and behavioural brood recognition cues (for a review see [30]), modulated further by brood developmental stage. Interestingly, with the exception of pupal intake in *C. smithi*, the ants did not distinguish between brood treatments, and brought in the live pathogen-exposed brood in equally high numbers as dead fungus or sham-treated brood (Table 1). This confirms a similar finding in another ant species [10] and seems to reveal the general pattern that, on one hand, the pathogen cannot manipulate its host in retrieving a higher proportion of contaminated versus healthy brood, and on the other hand, that potential repellent effects of the pathogen are likely overridden by the attractiveness of brood. This is despite the fact that social insects are capable of quickly detecting pathogen presence (ants: [10,31]; honeybees: [32]; termites: [33-35]). Brood being a strong elicitor for intake behaviour is also exploited, for example, by parasitic *Maculinea* butterflies, which morphologically and chemically mimic ant larvae and are picked up and brought into the nest [36].

Rapid detection and reaction to the fungal pathogen also occurred in our experiment. Brood grooming frequencies were significantly increased towards live fungus-exposed brood in species with cocooned pupae, *La. neglectus* and P. *punctata*, (Figure 1C,D) within the two days post exposure (i.e. before infection), whereas application of dead fungus was not enough to elicit this effect. This finding confirms previous reports of elevated grooming frequencies in other social insects, directed either towards live pathogen-exposed ant larvae [10], or adult nestmates in both ants [37] (but see [11]) and termites [12]. Currently it cannot be resolved, whether the other three species (*C. smithi, Li. humile* and *F. selysi*) did not show this adaptive behaviour due to a lack of pathogen detection or response. It seems that these species had an overall high grooming activity towards all brood, including the sham-treatment, which may suggest a constitutively high grooming level, acting as prophylactic defence (similar to [11]). Importantly, the observed upregulation of grooming directed towards pathogen-exposed brood in the two species with cocooned pupae documents that the presence of a silk cocoon around the pupae does not interfere with the ants’ capabilities to detect fungal conidiospores.

Sanitary brood care is not limited to the mechanical removal of infectious particles from the brood surface during allogrooming, and the following disinfection of those particles in the mouth of the cleaning individual and discarding in dump sites [9,38]. *La. neglectus* workers were recently found to further apply their antiseptic poison on their brood, thereby efficiently inhibiting germination of *Metarhizium* conidiospores [9]. The poison of other social Hymenoptera is also known to have antimicrobial properties and to be applied during nest hygiene (ants; subfamily Formicinae: [39], subfamily Myrmicinae: [40,41]; wasps: [42]; bees: [43]). It is thus likely that also the other ant species in our experiment may employ their poison in chemical brood disinfection. Ants further have evolved a unique gland–the metapleural gland–that produces potent antibiotic secretions, which can serve as antifungal defence [3,44]. All our study species possess metapleural glands, which may represent a second component of chemical surface disinfection complementing mechanical removal, though in *La. neglectus*, metapleural gland components do not seem to play a major role in protection of brood against *Metarhizium* fungus [9].

If these cleaning measures fail to prevent infection of the individual brood items, removal of the diseased brood from the colony is an effective way to limit disease transmission inside the colony. In fact, all ant species in our experiment showed hygienic behaviour, i.e. they removed assumedly infectious or infected brood from the brood chamber, thereby dooming this brood to death. This suggests that hygienic behaviour–so far mostly known from honeybees [2,10,14]–is a widespread behaviour also in ants. Species with naked pupae removed consistently more brood items (both larvae and pupae) treated with live fungus than sham control and dead fungus (Figures 2A,B and 3B; exceptions: ns trend in *Li. humile* larvae and sign. only against sham control in *F. selysi*). Species with cocooned pupae removed only their larvae in the same pattern, i.e. they also removed more live fungus-exposed larvae than either sham or dead fungus-treated larvae (Figure 2C,D). However, in the three species with obligatory or facultative cocoons, the cocooned pupae were all removed at equally low rates, independent of being exposed to the pathogen or control treatments (Figures 2C,D and 3C).

Differential removal patterns seem to represent an adaptive behaviour by the ants based on infection state (as in [10]). The risk of getting infected after exposure to live fungus, during the course of our experiment, was lowest in cocooned pupae, which were also removed less frequently, as compared to both, naked pupae and larvae (Figures 2, 3). By combining sanitary behaviours and selective removal of diseased brood, the ants successfully reduced the likelihood of the pathogen to self-replicate inside their nest. Only 5% of all fungal outgrowth thus occurred inside the nest, whereas 2/3 of the removed brood showed fungal outgrowth (black bars in Figures 2 and 3B,C).

Fungal outgrowth was not limited to the brood experimentally exposed to live fungal conidiospores, but also occurred on approximately 20% of the previously unexposed brood items, revealing disease transmission among brood (which we could monitor due to the colour marking of brood items). These high disease transmission rates explain, why also previously un-exposed control brood was removed at relatively high rates–particularly in the case of larvae (Figures 2 and 3B,C). It is interesting that the ants do not seem able to contain the transmission to healthy brood, and place all retrieved brood onto a common brood pile enabling disease contraction, but later are highly efficient in removing the infected brood.

In contrast to the high transmission of disease at the brood stage, cross infection of adult workers through contact to contaminated brood was practically absent (< 1%). This may result from both hygiene–including sanitary behaviours [5,45] and use of exocrine gland-derived antiseptics [3,9,39]–and a lower susceptibility due to a melanised and sclerotised cuticle [46]. Low rates of workers contracting the disease after contact to fungus-exposed brood or workers have also been reported for other ants and termites [10,12,45], whereas contact with sporulating cadavers led to high infection rates in ants [45]. This emphasizes the importance of hygienic brood removal before any visible fungal outgrowth. Indeed, in our experiment, we found that brood removal anticipated fungal outgrowth, and was performed approximately one to two days before fungal outgrowth (Figure 4), revealing that the ants are able to detect the infection state before infectious stages occur.

## Conclusions

Our study revealed that ants removed pathogen-exposed cocooned brood less frequently than exposed naked brood from their brood chamber, and that this behaviour seems adaptive given the lower risk of infection of the cocooned brood. Future work is required to disentangle the mechanism(s) underlying the observed protective effect of the pupal cocoon in ants. Among the proposed mechanisms are i) more frequent or efficient sanitary actions performed by the ants, ii) interference of the cocoon with fungal pathogenicity, or iii) a combination of both. Moreover, it remains to be tested if the pupal cocoon acts as a barrier restricting infectious fungal conidiospores to get in contact with the insect cuticle, which triggers germination [21]. Notably, the ant silk cocoon does not provide full protection against *Metarhizium* infection [47], in contrast to what has been found in other Hymenoptera (sawflies [48]). Differences in the level of the protection conveyed by cocoons may be due to differences in silk composition, as e.g. Lepidopteran silk proteins can have antimicrobial effects [49] with peptides in the cocoon acting as bacterial and fungal proteinase inhibitors [50]. In addition, antibiotic-producing bacteria can be integrated into the cocoon during weaving, as is the case in beewolves [51]. Regardless of the underlying mechanism, the presence of a silk cocoon seems to give the pupae some protection against fungal infection in the tested ant species. This may explain why at least a fraction of pupae have cocoons in species like *F. selysi* despite their production cost (estimated as a two days longer development of cocooned vs naked pupae; [26]). Our results thus support the hypothesis that *F. selysi* has a dual strategy, producing naked pupae that develop faster but run a higher risk of fungal infection, simultaneously with the more costly, slower developing but better protected cocooned pupae [26].

## Methods

### Host ants

The between-species comparison was performed on two ant species with cocooned pupae, *Platythyrea punctata* (Ponerinae) and *Lasius neglectus* (Formicinae), and two species with naked pupae, *Linepithema humile* (Dolichoderinae) and *Crematogaster smithi* (Myrmicinae). For the within-species analysis, we used *Formica selysi* (Formicinae), which is characterised by simultaneous presence of cocooned and naked pupae in the same nest [26]. All species were collected from 2005 to 2008 (*P. punctata*: Puerto Rico, Dominican Republic, Barbados, for collection details and authorisation see [52]; *La. neglectus*: France, Turkey, Spain (2 populations), Germany, Italy [53]; *C. smithi*: USA [23]; *Li. humile*: Spain [54]; *F. selysi:* Switzerland [27]). For each species, a minimum of 12 different colonies was collected and used as replicates in the experiments, with the exception of *F. selysi*, for which only 10 colonies were obtained. For the unicolonial species *La. neglectus* and *Li. humile*, replicates consisted of different nests collected either from the same (*Li. humile*) or different supercolonies (*La. neglectus*, see [53]). All experiments comply with European laws.

### Fungal pathogen and exposure

We exposed brood items to conidiospore suspensions (0.3 μl of 1 × 10^9^ conidiospores/ml in sterile 0.05% Triton X-100; Sigma) of the entomopathogenic fungus *Metarhizium brunneum* (strain KVL 03–143 / Ma 275; previously referred to as *M. anisopliae*, but now separated as a sister species; [55]). *M. brunneum* has a worldwide distribution and is a natural pathogen of ants [55], yet the used strain was isolated from Austria and therefore lacks a local co-evolutionary history with all studied host ant populations. Conidiospores were either alive (live fungus; 98% germination rate) or killed by UV irradiation (312 nm with 6*15 W for 1 h, see [31]; dead fungus; 0% germination). Sham treatment consisted of application of the same amount of sterile 0.05% Triton X only.

### Experimental design

Three groups of brood treated with either sham control, dead or live fungus, were simultaneously added to five individually colour marked (Edding 780) ant workers, placed in experimental nests containing a brood chamber (as in [31]; n = 12 replicates per species). In the between-species comparison, each group of brood items consisted of two larvae (excluding the smallest developmental stages) and two pupae (exceptions: four *P. punctata* replicates contained only one pupa and two larvae and all *Li. humile* replicates had only one larva and two pupae; to take these differences into account, data were standardised to the level of a single brood item prior to statistical analysis). For the within-species analysis of *F. selysi,* each group of brood items consisted of two naked and two cocooned pupae. All brood items of the same treatment were equally colour marked (but colour use randomised over replicates to prevent an observer bias) and placed on 1 × 1 cm filter paper, presented equidistantly to the brood chamber.

### Behavioural observations

In the first two days of the experiment, we observed each replicate 5 to 10 times per day (mean ± s.e.m., 6.2 ± 0.4 times) by scan sampling of each individual ant worker and brood item (as described in [10]) to obtain detailed data on brood intake into the brood chamber and brood grooming by the workers, as *Metarhizium* conidiospores can still be removed by grooming in the first 48 h after exposure before they attach firmly to the host cuticle [56]. On each day of the experiment (days 1–12), brood location (inside/outside the brood chamber), brood fungal outgrowth, as well as worker survival was determined. Among the pupae taken into the brood chamber, 14% eclosed to adult workers during the course of the experiment (total 41/291, 14/113 naked and 27/178 cocooned pupae). Proportions were equal for naked and cocooned pupae, as well as across treatments, and removed cocoons were placed outside the brood chamber independent of treatment. Data of eclosed pupae were censored at the day of eclosion. Workers dying within the experimental period were collected, surface sterilized [57] and transferred to Petri dishes containing damp filter paper (21 ± 3°C) to determine *Metarhizium* infection of the corpses (hyphal outgrowth and conidiospore production occurring within three weeks).

### Statistical analyses

All statistical analyses were carried out using R [58].

#### Brood grooming

Brood grooming frequencies were calculated for each brood treatment group per replicate as the number of grooming events divided by the number of scan samplings and standardized to the number of workers still alive and to a single brood item per day and then summed for the two days. Normalised (square-root transformed) data were analysed in a Linear Mixed Model (LMM; package “lme4” [59], and “multcomp”[60]) to test for the effect of brood type (between-species: larvae vs pupae, within-species: naked vs cocooned pupae) and brood treatment (sham control, dead or live fungus) for each species. Colony origin of brood and of workers was included as random factors. None of the interactions were significant, and *P* values thus not reported.

#### Brood intake and removal

Location of the brood (intake to the brood chamber and subsequent removal) was analysed as a time-course analysis using Cox regression (mixed-effects model; package “coxme” [61]) with the colony origin of brood and workers as random factors. The effect of treatment on brood location was tested at the species level separately for each brood type. Cox mixed-effects models cannot run on completely censored data (pers. com. Terry Therneau, developer of the R package “coxme”). This occurred twice in our dataset, as none of the sham-treated larvae of *C. smithi* and pupae of *P. punctata* were removed from the brood chamber. To avoid complete censoring, we changed the status of a single individual to “removed” on the last day of observation in these two cases. The robustness of this procedure was investigated by modelling the unaltered data with Kaplan-Meier survival analysis with Log-rank tests (package “survival” [62]), and gave consistent results. We used the method of Westfall to adjust the family wise error rate for posthoc multiple comparisons between treatment levels [60]. Possible differences in the intake and removal of brood depending on their type were analysed at the species level across all treatments using a Pearson’s χ^2^-test with Yates’ continuity correction.

#### Fungal outgrowth

We used Pearson’s correlation analysis to examine the relationship between the mean day of brood removal and fungal outgrowth for all brood items showing fungal outgrowth across the five study species. The mean days were calculated for each treatment within the corresponding brood type (larvae, naked and cocooned pupae), averaged over all species. We used Pearson’s χ^2^-test with Yates’ continuity correction to assess differences in fungal outgrowth between all removed brood and all brood remaining inside the nest chamber across all treatments and ant species, and also to compare fungal outgrowth of live fungus-exposed brood depending on brood type, by first assessing overall significance (3 × 2 contingency table) followed by all pairwise comparisons (2 × 2), corrected by Bonferroni procedure of multiple testing.

## Availability of supporting data

The data sets supporting the results of this article are available in the DRYAD repository (http://doi.org/10.5061/dryad.nc0gc).

## Competing interests

The authors declare that they have no competing interests.

## Authors’ contributions

LVU, MC, JH and SC conceived the experiments; LVU, MC and SC collected ants; ST, LVU and SC performed the experiments; ST and LVU performed the statistical analysis; ST, LVU and SC wrote the paper. All authors read and acknowledged the final version of the manuscript.

## References

[B1] Cremer S Armitage SAO Schmid-Hempel P Social immunity Curr Biol 2007 17 16 R693 R702 10.1016/j.cub.2007.06.008 17714663

[B2] Wilson-Rich N Spivak M Fefferman NH Starks PT Genetic, individual, and group facilitation of disease resistance in insect societies Annu Rev Entomol 2009 54 405 423 10.1146/annurev.ento.53.103106.093301 18793100

[B3] Fernández-Marín H Zimmerman JK Rehner SA Wcislo WT Active use of the metapleural glands by ants in controlling fungal infection Proc R Soc B 2006 273 1689 1695 10.1098/rspb.2006.3492 16769642 PMC1634922

[B4] Naug D Camazine S The role of colony organization on pathogen transmission in social insects J Theor Biol 2002 215 4 427 439 10.1006/jtbi.2001.2524 12069487

[B5] Oi DH Pereira RM Ant behavior and microbial pathogens (Hymenoptera: Formicidae) Fla Entomol 1993 76 63 73 10.2307/3496014

[B6] Noirot C Pasteels JM Ontogenetic development and evolution of the worker caste in termites Experientia 1987 43 8 851 952 10.1007/BF01951642

[B7] Thompson PR Hepburn HR Changes in chemical and mechanical properties of honeybee (*Apis mellifera adansonii* L.) cuticle during development J Comp Physiol B 1978 126 3 257 262 10.1007/BF00688935

[B8] Patterson RS Briano JA Potential of three biological control agents for suppression of *Solenopsis invicta*, the red imported fire ant Proceedings of the 1st International Conference on Insect Pests on Urban Environments 1993 Exeter, UK 35 43

[B9] Tragust S Mitteregger B Barone V Konrad M Ugelvig LV Cremer S Ants disinfect fungus-exposed brood by oral uptake and spread of their poison Curr Biol 2013 23 1 76 82 10.1016/j.cub.2012.11.034 23246409

[B10] Ugelvig LV Kronauer DJC Schrempf A Heinze J Cremer S Rapid anti-pathogen response in ant societies relies on high genetic diversity Proc R Soc B 2010 277 1695 2821 2828 10.1098/rspb.2010.0644 20444720 PMC2981995

[B11] Reber A Purcell J Buechel SD Buri P Chapuisat M The expression and impact of antifungal grooming in ants J Evol Biol 2011 24 954 956 10.1111/j.1420-9101.2011.02230.x 21306465

[B12] Rosengaus RB Maxmen AB Coates LE Traniello JFA Disease resistance: a benefit of sociality in the dampwood termite *Zootermopsis angusticollis* (Isoptera: Termopsidae) Behav Ecol Sociobiol 1998 44 2 125 134 10.1007/s002650050523

[B13] Yanagawa A Shimizu S Resistance of the termite, *Coptotermes formosanus* Shiraki to *Metarhizium anisopliae* due to grooming BioControl 2007 52 75 85 10.1007/s10526-006-9020-x

[B14] Rothenbuhler WC Thompson VC Resistance to american foulbrood in honey bees. I. Differential survival of larvae of different genetic lines J Econ Entomol 1956 49 470 475

[B15] Armitage SAO Fernández-Marín H Wcislo WT Boomsma JJ An evaluation of the possible adaptive function of fungal brood covering by attine ants Evolution 2012 66 6 1966 1975 10.1111/j.1558-5646.2011.01568.x 22671560

[B16] Baroni Urbani C Bolton B Ward PS The internal phylogeny of ants (Hymenoptera: Formicidae) Syst Entomol 1992 17 301 329 10.1111/j.1365-3113.1992.tb00553.x

[B17] Danks HV The roles of insect cocoons in cold conditions Eur J Entomol 2004 101 3 433 437

[B18] Ayasse M Paxton R Hilker M, Meiners T Brood protection in social insects Chemoecology of insect eggs and egg deposition 2002 Berlin: Blackwell 117 148

[B19] Hughes WOH Petersen KS Ugelvig LV Pedersen D Thomsen L Poulsen M Boomsma JJ Density-dependence and within-host competition in a semelparous parasite of leaf-cutting ants BMC Evol Biol 2004 4 45 10.1186/1471-2148-4-45 15541185 PMC535352

[B20] Reber A Chapuisat M Diversity, prevalence and virulence of fungal entomopathogens in colonies of the ant *Formica selysi* Insectes Soc 2012 59 2 231 239 10.1007/s00040-011-0209-3

[B21] Castrillo LA Roberts DW Vandenberg JD The fungal past, present, and future: germination, ramification, and reproduction J Invertebr Pathol 2005 89 1 46 56 10.1016/j.jip.2005.06.005 16039305

[B22] Heller NE Colony structure in introduced and native populations of the invasive argentine ant, *Linepithema humile* Insectes Soc 2004 51 4 378 386 10.1007/s00040-004-0770-0

[B23] Oettler J Schmitt T Herzner G Heinze J Chemical profiles of mated and virgin queens, egg-laying intermorphs and workers of the ant *Crematogaster smithi* J Insect Physiol 2008 54 4 672 679 10.1016/j.jinsphys.2008.01.004 18321526

[B24] Schilder K Heinze J Hölldobler B Colony structure and reproduction in the thelytokous parthenogenetic ant *Platythyrea punctata* (F. Smith) (Hymenoptera, Formicidae) Insectes Soc 1999 46 2 150 158 10.1007/s000400050126 10564455

[B25] Van Loon AJ Boomsma JJ Andrasvalvy A A new polygynous *Lasius* species (Hymenoptera; Formicidae) from central Europe I. Description and general biology Insectes Soc 1990 37 4 348 362 10.1007/BF02225997

[B26] Purcell J Chapuisat M The influence of social structure on brood survival and development in a socially polymorphic ant: insights from a cross-fostering experiment J Evol Biol 2012 25 11 2288 2297 10.1111/j.1420-9101.2012.02607.x 22998635

[B27] Chapuisat M Bocherens S Rosset H Variable queen number in ant colonies: no impact on queen turnover, inbreeding, and population genetic differentiation in the ant *Formica selysi* Evolution 2004 58 5 1064 1072 15212387 10.1111/j.0014-3820.2004.tb00440.x

[B28] Brian MV Larval recognition by workers of the ant *Myrmica* Anim Behav 1975 23 745 756 10.1016/s0003-3472(73)80093-4 4777198

[B29] Lenoir A Brood retrieving in the ant, *Lasius niger* L Sociobiology 1981 6 153 178

[B30] Vander Meer RK Morel L Trager JC Brood pheromones in ants Advances in myrmecology 1988 Leiden, The Netherlands: Brill 419 513

[B31] Ugelvig LV Cremer S Social prophylaxis: group interaction promotes collective immunity in ant colonies Curr Biol 2007 17 22 1967 1971 10.1016/j.cub.2007.10.029 17980590

[B32] Evans JD Spivak M Socialized medicine: individual and communal disease barriers in honey bees J Invertebr Pathol 2010 103 Suppl 1 S62 S72 19909975 10.1016/j.jip.2009.06.019

[B33] Mburu DM Ochola L Maniania NK Njagi PGN Gitonga LM Ndung’u MW Wanjoya AK Hassanali A Relationship between virulence and repellency of entomopathogenic isolates of *Metarhizium anisopliae* and *Beauveria bassiana* to the termite *Macrotermes michaelseni* J Insect Physiol 2009 55 9 774 780 10.1016/j.jinsphys.2009.04.015 19442668

[B34] Yanagawa A Fujiwara-Tsujii N Akino T Yoshimura T Yanagawa T Shimizu S Odor aversion and pathogen-removal efficiency in grooming behavior of the termite *Coptotermes formosanus* PloS ONE 2012 7 10 e47412 10.1371/journal.pone.0047412 23077609 PMC3471821

[B35] Yanagawa A Yokohari F Shimizu S The role of antennae in removing entomopathogenic fungi from cuticle of the termite, *Coptotermes formosanus* J Insect Sci 2009 9 6 1 9 10.1673/031.009.0601 PMC3011873 19611249

[B36] Akino T Knapp JJ Thomas JA Elmes GW Chemical mimicry and host specificity in the butterfly *Maculinea rebeli*, a social parasite of *Myrmica* ant colonies Proc R Soc B 1999 266 1427 1419 1426 10.1098/rspb.1999.0796

[B37] Walker TN Hughes WOH Adaptive social immunity in leaf-cutting ants Biol Lett 2009 5 446 448 10.1098/rsbl.2009.0107 19411266 PMC2781909

[B38] Little AEF Murakami T Mueller UG Currie CR The infrabuccal pellet piles of fungus-growing ants Naturwissenschaften 2003 90 558 562 10.1007/s00114-003-0480-x 14676952

[B39] Graystock P Hughes WOH Disease resistance in a weaver ant, *Polyrhachis dives*, and the role of antibiotic-producing glands Behav Ecol Sociobiol 2011 65 2319 2327 10.1007/s00265-011-1242-y

[B40] Storey GK Vander Meer RK Boucia DG McCoy CW Effect of fire ant (*Solenopsis invicta*) venom alkaloids on the in vitro germination and development of selected entomogenous fungi J Invertebr Pathol 1991 58 88 95 10.1016/0022-2011(91)90166-N

[B41] Vander Meer RK Ant queens deposit pheromones and antimicrobial agents on eggs Naturwissenschaften 1995 82 93 95 10.1007/BF01140150

[B42] Baracchi D Mazza G Turillazzi S From individual to collective immunity: the role of the venom as antimicrobial agent in the Stenogastrinae wasp societies J Insect Physiol 2011 58 188 193 22108024 10.1016/j.jinsphys.2011.11.007

[B43] Baracchi D Francese S Turillazzi S Beyond the antipredatory defence: honey bee venom function as a component of social immunity Toxicon 2011 58 550 557 10.1016/j.toxicon.2011.08.017 21925197

[B44] Yek SH Mueller UG The metapleural gland of ants Biol Rev 2011 86 4 774 791 10.1111/j.1469-185X.2010.00170.x 21504532

[B45] Hughes WOH Eilenberg J Boomsma JJ Trade-offs in group living: transmission and disease resistance in leaf-cutting ants Proc R Soc B 2002 269 1502 1811 1819 10.1098/rspb.2002.2113 12350269 PMC1691100

[B46] Moret Y Moreau J The immune role of the arthropod exoskeleton Invertebrate Surviv J 2012 9 200 206

[B47] Fountain T Hughes WOH Weaving resistance: silk and disease resistance in the weaver ant *Polyrhachis dives* Insectes Soc 2011 58 453 458 10.1007/s00040-011-0162-1

[B48] Führer E Rosner S Schmied A Wegensteiner R Studies on the significance of pathogenic fungi in the population dynamics of the lesser spruce sawfly, *Pristiphora abietina* Christ. (Hym., Tenthredinidae) J Appl Entomol 2001 125 235 242 10.1046/j.1439-0418.2001.00529.x

[B49] Korayem AM Hauling T Lesch C Fabbri M Lindgren M Loseva O Schmidt O Dushay MS Theopold U Evidence for an immune function of lepidopteran silk proteins Biochem Biophys Res Commun 2007 352 2 317 322 10.1016/j.bbrc.2006.11.022 17126296

[B50] Nirmala X Kodrik D Zurovec M Sehnal F Insect silk contains both a kunitz-type and a unique kazal-type proteinase inhibitor Eur J Biochem 2001 268 7 2064 2073 10.1046/j.1432-1327.2001.02084.x 11277929

[B51] Kroiss J Kaltenpoth M Schneider B Schwinger MG Hertweck C Maddula RK Strohm E Svatos A Symbiotic streptomycetes provide antibiotic combination prophylaxis for wasp offspring Nat Chem Biol 2010 6 4 261 263 10.1038/nchembio.331 20190763

[B52] Kellner K Heinze J Absence of nepotism in genetically heterogeneous colonies of a clonal ant Ethology 2011 117 6 556 564 10.1111/j.1439-0310.2011.01910.x

[B53] Ugelvig LV Drijfhout FP Kronauer DJC Boomsma JJ Pedersen JS Cremer S The introduction history of invasive garden ants in Europe: integrating genetic, chemical and behavioural approaches BMC Biol 2008 6 11 10.1186/1741-7007-6-11 18302731 PMC2292682

[B54] Vogel V Pedersen JS Giraud T Krieger MJB Keller L The worldwide expansion of the argentine ant Divers Distrib 2010 16 1 170 186 10.1111/j.1472-4642.2009.00630.x

[B55] Bischoff JF Rehner SA Humber RA A multilocus phylogeny of the *Metarhizium anisopliae* lineage Mycologia 2009 101 4 512 530 10.3852/07-202 19623931

[B56] Hänel H The life cycle of the insect pathogenic fungus *Metarhizium anisopliae* in the termite *Nasutitermes exitiosus* Mycopathologia 1982 80 137 145 10.1007/BF00437576

[B57] Lacey LA Brooks WM Lacey LA Initial handling and diagnosis of diseased insects Manual of techniques in insect pathology 1997 London: Academic Press 1 16

[B58] R Development Core Team R: a language and environment for statistical computing 2012 Vienna, Austria: R Foundation for Statistical Computing

[B59] Bates D Maechler M Bolker Blme4: linear mixed-effects models using S4 classes. R package version 0.999375-42 2011 http://cran.r-project.org/package=lme4

[B60] Bretz F Hothorn T Westfall P Multiple comparisons using R 2011 Boca Raton, FL: CRC Press

[B61] Therneau T Coxme: mixed effects cox models. R package version 2.2-3 2012 http://cran.r-project.org/package=coxme

[B62] Therneau T A package for survival analysis in S. R package version 2.36-14 2012 http://CRAN.R-project.org/package=survival

